# Psychological wellbeing among carers of children and adolescents with physical disabilities in North-Eastern Tanzania—a cross-sectional survey study

**DOI:** 10.3389/fpubh.2024.1437716

**Published:** 2024-10-09

**Authors:** Elia Asanterabi Swai, Haleluya Imanueli Moshi, Sia Emmanueli Msuya, Ann Sörlin, Klas-Göran Sahlen, Marie Lindkvist

**Affiliations:** ^1^Department of Epidemiology and Global Health, Umeå University, Umeå, Sweden; ^2^Department of Physiotherapy, Kilimanjaro Christian Medical University College, Moshi, Tanzania; ^3^Department of Community Medicine and Rehabilitation, Umeå University, Umeå, Sweden; ^4^Department of Physiotherapy, Kilimanjaro Christian Medical Centre, Moshi, Tanzania; ^5^Community Health Department, Institute of Public Health, Kilimanjaro Christian Medical University College, Moshi, Tanzania; ^6^Department of Epidemiology and Biostatistics, Institute of Public Health, Kilimanjaro Christian Medical University College, Moshi, Tanzania; ^7^Department of Community Medicine, Kilimanjaro Christian Medical Centre, Moshi, Tanzania

**Keywords:** physical disability, carers, children, adolescents, Tanzania, psychological wellbeing, anxiety, depression

## Abstract

**Introduction:**

Caring for physically disabled children, particularly in contexts where resources are often insufficient or absent, exposes primary carers to significant psychological strain. The lack of structured mechanisms to address this psychological burden poses a considerable threat to the wellbeing of both carers and the children with disabilities under their care. However, research on the psychological wellbeing of carers in Tanzania is scarce. This study aimed to evaluate the psychological wellbeing and symptoms of anxiety and depression, along with associated factors, of carers of children, and adolescents with physical disabilities in the Kilimanjaro region.

**Methods:**

A cross-sectional survey was conducted from November 2020 to June 2021 in the Kilimanjaro region, involving 212 carers. The Swahili versions of the WHO-5 Wellbeing Index and Hopkins Symptoms Check List-25 were used to assess psychological wellbeing and symptoms of anxiety and depression. Data were analysed using IBM SPSS Statistics V.28. Multivariable linear and binary logistic regression were used to assess the associations.

**Results:**

More than four fifths (81%) of the carers were parents, and most of these parents were mothers (92%). Of the 212 carers, more than half (51%) exhibited poor psychological wellbeing, 42% had symptoms of anxiety, and 38% symptoms of depression. Notably, poor psychological wellbeing was significantly associated with symptoms of anxiety and depression. Results of a multivariable regression analysis indicated that extended family support was associated with better psychological wellbeing and lower odds of symptoms of anxiety and depression. A higher estimated monthly income was associated with better psychological wellbeing, and lower odds of symptoms of depression. Access to rehabilitation for the child was associated with improved psychological wellbeing and decreased likelihood of symptoms of anxiety.

**Conclusion:**

Many carers of children, and adolescents with physical disabilities in Tanzania are at high risk of having poor psychological wellbeing, and symptoms of anxiety and depression. Support from extended-family networks is crucial in promoting good psychological wellbeing. The government in Tanzania should improve rehabilitation services, especially in disadvantaged rural areas, and integrate mental-health screening for carers in both rehabilitation programmes and primary healthcare.

## 1 Introduction

Psychological wellbeing is a fundamental pillar of overall human health (1, 2) that relates to feeling satisfied with life and being able to function effectively (3). Taking care of children, and adolescents with physical disabilities can be challenging for primary carers (4, 5). In this study, a carer is defined as a close family member who is responsible for caring for a child or adolescent in all aspects of life, and who does not receive financial compensation for doing so (4, 6). Children, and adolescents with physical disabilities are in this study operationalised as individuals aged between 2 and 18 with lifelong structural or functional impairments (7–9). In children and adolescents, physical disabilities can occur before birth (congenital), such as spina bifida, muscular dystrophy, or be acquired during or after birth due to injury, such as cerebral palsy, amputation, or spinal cord injury (10). These impairments may or may not be accompanied by cognitive deficits, and can make it hard for them to do things like move around the world and take part in different aspects of life (7). According to the Convention on the Rights of Persons with Disabilities, disability results from the interaction between impairments and societal barriers (7). Caregiving responsibilities often lead to significant socioeconomic difficulties, which can affect carers’ psychological wellbeing (11). These difficulties are experienced to a greater extent in low- and middle-income countries (LMICs) than high-income countries (HICs) due to differences in policies, funds allocated for children living with physical disabilities, limited healthcare systems, and poor linkage with communities (12).

Carers of children and adolescents with physical disabilities (the majority being women) often reports symptoms of anxiety and depression (13–18). According to the World Health Organization (WHO), anxiety and depression are common mental-health issues that have a higher prevalence among women (19, 20). They can affect individuals who deal with challenging situations, such as caring for a child or adolescent with physical disability; doing so in a low-income countries (LICs) setting such as Tanzania can exacerbate this condition. Previous research has found that depression, and overall health status of cares of children with disabilities, is linked to the family income of carers and type and severity of disability of the individual being cared for (16, 21). Therefore, promoting the wellbeing of carers of children, and adolescents with physical disabilities is an important public-health concern.

In 2019, around 4% of the global population experienced an anxiety disorder (20, 22). However, rates of anxiety vary worldwide, with higher prevalence in Latin America, the Caribbean, North America, and Western Europe than in South Asia and sub-Saharan Africa (SSA) (22). A review of data from 33 LMICs, which included 51 studies, found that anxiety and depression were the predominant psychological conditions among family carers (23). The reported rates of anxiety and depression among carers varied significantly, ranging from 52% to 94% (23).

An analysis of the World Health Survey’s (WHS) cross-sectional data from 47 LMICs revealed a 19.1% prevalence of anxiety symptoms among informal carers (24). Moreover, in that study carers were almost twice as likely to experience anxiety as non-carers, and the anxiety rate was higher for upper-middle income countries (UMICs) compared to the rest of LMICs. Similarly, depression symptoms were found in 10% of carers compared to 6.4% of non-carers, with higher rates observed in HICs compared to LMICs (25).

Research on carers’ psychological wellbeing in Tanzania is scant, particularly for those caring for children, adolescents, or persons with disabilities. Previous research conducted in Tanzania has explored the prevalence of anxiety and depression among diverse populations beyond carers of children, and adolescents with physical disabilities and has yielded varying insights. For example, in one study in north-west Tanzania, 33% of adolescent girls and young women reported symptoms of anxiety, while 36% exhibited signs of depression (26). Similarly, a survey conducted in the Dar es Salaam region of Tanzania showed rates of 19% for anxiety and 20% for depression among young men (27). Mental-health interventions specifically for the carers of children, and adolescents with physical disabilities are notably lacking in the mainstream healthcare system in Tanzania, and need to be integrated and strengthened (2). Not attending to mental-health issues can negatively impact both how carers cope with situations and overall wellbeing (28). Poor psychological wellbeing, leading to anxiety and/or depression, poses a significant threat to the wellbeing of the children who receive care, the carers themselves, and other members of the family (29).

Factors associated with psychological wellbeing of carers of children, and adolescents with disabilities such as income status, severity of disability, and the presence of support are often emphasised in research (14, 16, 30, 31). Literature from HICs has found that children’s visual impairment, frequent hospital admissions, and visits to emergency units are associated with carers’ depression and anxiety (32). In middle-income countries (MICs). In the middle-income countries (MICs), low levels of depression are linked with strong family relationships, a better barrier-free environment, and home ownership, while frequent mood swings in children and higher education among carers increase the likelihood of depression. Similarly, carers marital status, and low education level have shown association with anxiety, while carers’ physical problems, and the type of child’s impairment are linked to depression (14, 16, 30). Additionally, literature from African settings has shown that carer depression is associated with sex, age, proximity to health facilities, and occupation (13).

Carers play a crucial role in ensuring the wellbeing of vulnerable physically disabled children. In LICs, the burden of care falls on the family, because there is no governmental system or structure in place to take care of children and adolescents with disabilities (23). In Tanzania, as in other SSA countries, it been observed that mothers often take on the caregiving responsibilities for these children, while fathers typically act as economic providers (33). Providing care for children with physical disabilities can be mentally and physically exhausting, especially when resources are scarce or absent, health systems do not work, and support structures are not in place (28, 34, 35). Furthermore, over time there is a tendency for the care of children and adolescents with physical disabilities to be left entirely to the nuclear family, or to the mother alone (36). This strain can significantly affect these individuals in terms of their mental health and overall psychological wellbeing (4, 21). Therefore, psychological support for carers in such settings is essential.

Documenting the psychological status and symptoms of anxiety and depression can highlight the need for essential public-health interventions in the Tanzanian context. Research in this area is crucial to enacting revision of actions and policy relating to mental health. The findings of this study will contribute to the body of knowledge on psychological wellbeing, particularly symptoms of anxiety and depression, among carers of children and adolescents with physical disabilities from LIC settings. Additionally, documenting the mental health of this population is crucial for informing Sustainable Development Goal 3, which aims to ensure healthy lives and promote wellbeing for all at all ages.

Screening tools that have been validated in Swahili have enhanced how psychological wellbeing, anxiety, and depression are assessed in Tanzania (26, 27, 37–40). The Hopkins Symptoms Check List-25 (HSCL-25) and WHO-5 Wellbeing Index, among others, are used to screen for anxiety and depression on a population scale (37, 38). However, research on the psychological burden faced by carers of children with physical disabilities remains limited in Tanzania, and specific research on anxiety and depression among carers in this context has not been performed. Literature on mental-health support for carers is also notably scarce in Tanzania, posing a challenge to the goal of prioritising mental health and wellbeing for all (2, 8). The aims of this study are twofold: Firstly, to evaluate the psychological wellbeing of and symptoms of anxiety and depression among carers of children and adolescents with physical disabilities in the Kilimanjaro region. Secondly, to assess the factors associated with psychological wellbeing and symptoms of anxiety and depression in this population.

## 2 Materials and methods

### 2.1 Study design and settings

**Figure 1:**
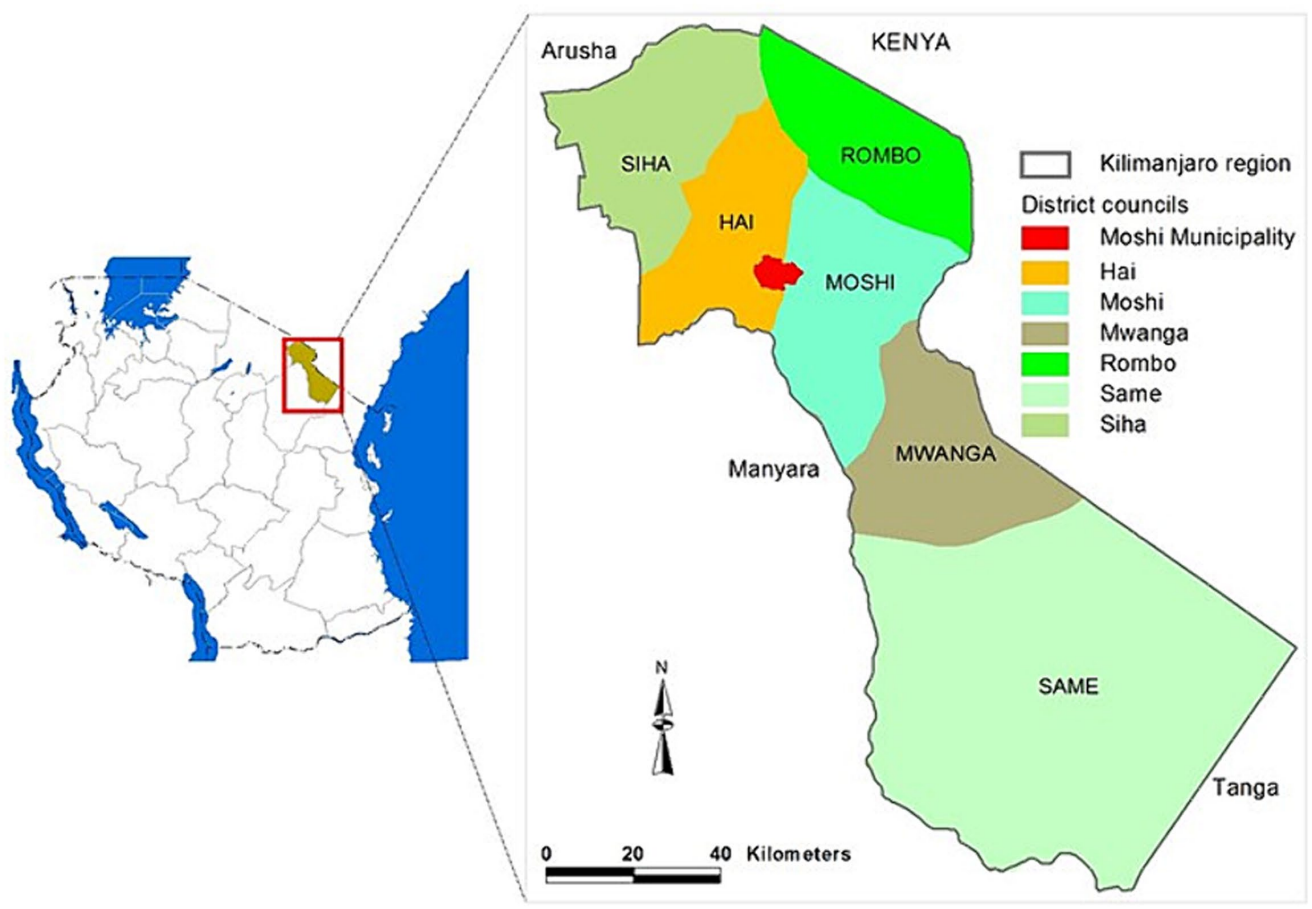
A map showing the administrative structures of the study area. Reprinted with permission from (42), licensed under CC BY 4.0, https://doi.org/10.4236/ojss.2014.413044.

This study was conducted between November 2020 and June 2021 in Kilimanjaro, one of Tanzania’s 31 administrative regions. The study used a cross-sectional household survey design, and some of its findings were previously published (41). Kilimanjaro has a population density of 141 people per square kilometre. Located in north-eastern Tanzania, it shares borders with Kenya to the north-east, the Tanga region to the south, the Manyara region to the south-west, and the Arusha region to the west (see Figure 1) (42). A significant portion of its population lives in rural areas.

The region is divided into seven district councils: Moshi Municipal, Moshi Rural, Hai, Rombo, Mwanga, Same, and Siha. These districts are then divided into wards, which further break down into streets in urban areas or villages in rural ones. The number of wards ranges from 17 in Hai and Siha to 32 in Same. Additionally, rural villages are subdivided into hamlets, and these hamlets into 10-cells (which consist of 10 households), which are the smallest administrative units. To ensure representation and reduce bias in the study, districts were chosen as the primary sampling unit.

According to the 2022 census of Tanzania’s population and households, Kilimanjaro had a total of 461 healthcare facilities, with only 7% categorised as hospitals offering referral or specialised services (43). Referring to the 2014–2019 staffing levels under the Tanzanian Ministry of Health (44), rehabilitation services for children and adolescents with disabilities are available at district, regional, and tertiary hospitals, which constitute less than 10% of the health facilities in the study area.

### 2.2 Study population and sampling

The study participants were adult primary carers, aged 18 and above, who are responsible for children and adolescents with physical disabilities. We included the primary carers of children and adolescents aged 2 to 18 with physical disabilities. It was a requirement that participants be able to communicate fluently in the Swahili language. Carers of children with acute medical conditions, in addition to their disabilities, who needed an urgent medical attention were excluded from the study and advised to seek treatment at a nearby healthcare facility.

**Figure 2:**
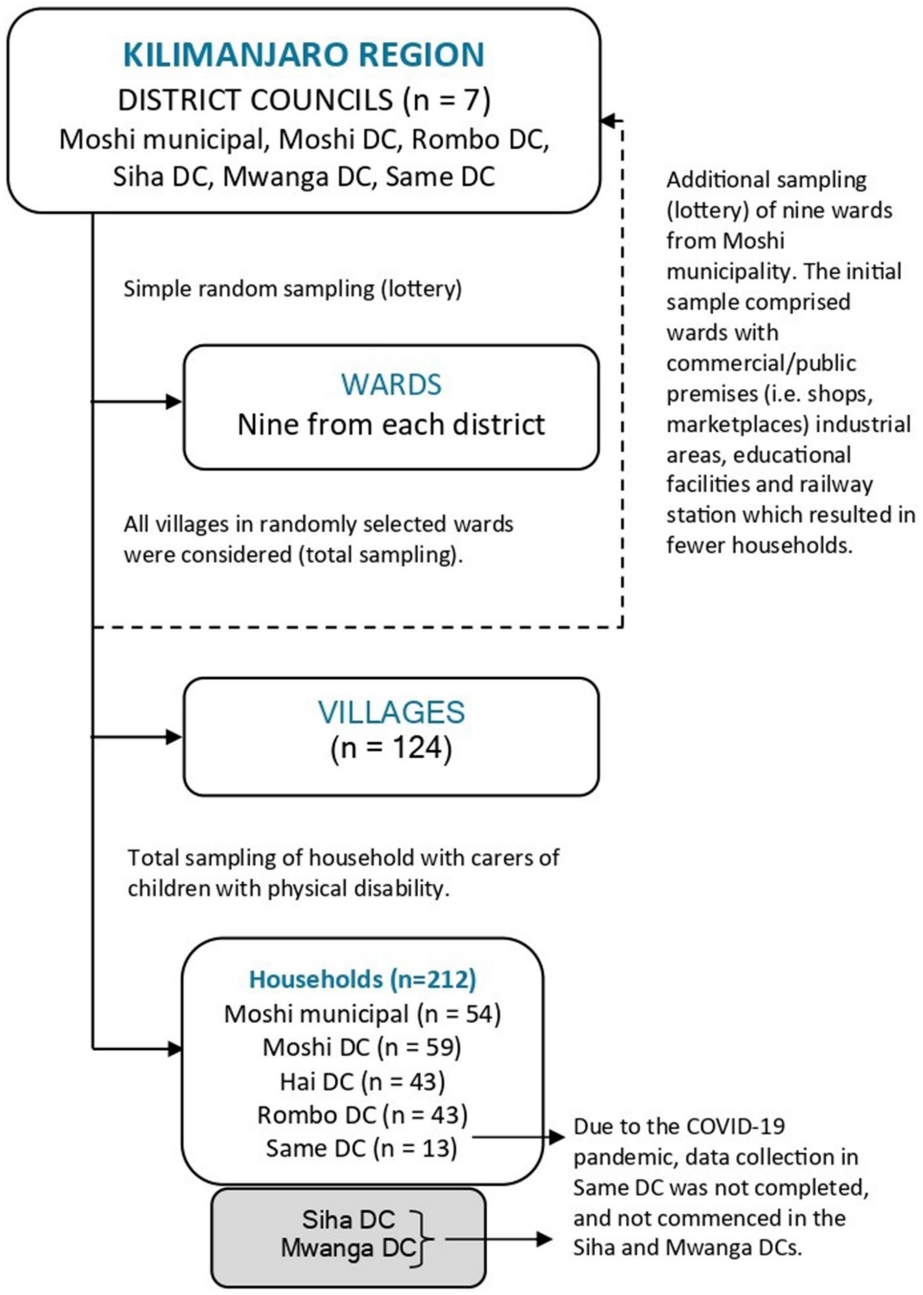
A flowchart illustrating the sampling procedure. DC, District Council.

Household sampling was conducted in three stages, using a simple random technique (lottery) and total sampling, as illustrated in Figure 2. All seven primary administrative units of the Kilimanjaro region, known as district councils, were considered. From each district, nine wards (district subdivisions) were randomly selected. All villages in the randomly selected wards were included; on the village level, all identified households with children and adolescents with physical disabilities were involved.

An additional sample of nine wards was drawn in Moshi municipality using the same procedure. This was done in order to increase the representation of participants from Moshi, because the initial sample was mostly comprised of public or business premises, such as industrial areas, a railway station, and educational institutions. Additionally, fieldwork and data-collection setbacks were encountered in the Siha, Mwanga, and Same districts due to COVID-19.

### 2.3 Data-collection tools

#### 2.3.1 Descriptive characteristics

Descriptive characteristics, including sociodemographic and caregiving-related information, were collected using a questionnaire that has been previously described (41, 45). These questionnaire-items were adapted from a local community survey questionnaire undertaken by the Institute of Public Health of the Kilimanjaro Christian Medical University College (45). Moreover, this tool had been previously peer-reviewed by experts in rehabilitation, public health and epidemiology. Questions regarding the characteristics of disabilities, such as activity limitations and restrictions on participation in schooling, were formulated based on the International Classification of Functioning Disability and Health (ICF) framework (7).

#### 2.3.2 Psychological wellbeing

The subjective psychological wellbeing of the carers was assessed using the WHO-5 Wellbeing Index, which is one of the most widely used research questionnaires worldwide (46). It was first introduced during the 1998 WHO Regional Office for Europe Congress (47), and has since been translated into more than 30 languages, including Swahili (40, 46–48). The Swahili version of the WHO-5 has been found to have good psychometric properties (40), and overall is reported to be sensitive and specific enough for screening depression (46, 49). The instrument assesses the state of wellbeing over the preceding 2 weeks.

The WHO-5 comprises five items, detailed in Supplementary Table S1. Response options range from 0 to 5, with 0 indicating ‘at no time’ and 5 indicating ‘all the time’. In this study, the WHO-5 was calculated as the sum of the scores of the responses, ranging from 0 (indicating the lowest possible wellbeing) to 25 (the highest possible wellbeing). For the purposes of comparison and interpretation, the raw scores were converted to a 0–100 scale, with lower scores indicating worse wellbeing. A score of ≤50 can indicate poor wellbeing, suggesting further screening for depression (50).

#### 2.3.3 Symptoms of anxiety and depression

Symptoms of anxiety and depression were assessed using the Swahili-language version of the HSCL-25. This instrument is widely used to evaluate symptoms of anxiety and depression (51, 52), and has been translated into and validated in various languages, including Swahili (37, 38). The Swahili version of the HSCL-25 has been previously used in Tanzania and is considered to be valid and reliable (37, 38, 53). The HSCL-25 involves 25 items (Supplementary Table S2) that relate to symptoms experienced over the preceding month: the first 10 items relate to anxiety, and the following 15 to depression. The response options are “Not at all,” “A little,” “Quite a bit,” and “Extremely,” and are scored 1 to 4, respectively. The final score is derived by summing the scores of all items in respective symptoms of anxiety and/or depression and dividing by the number of items (scores range from 1.00 to 4.00, 10 items for anxiety and 15 items for depression). A score of ≥1.75 on those two scales are indicative of symptoms of anxiety and/or depression (51).

### 2.4 Data-collection procedures

Data collection was conducted by two research assistants, who underwent rigorous pretraining on the study protocols, and first author. We used the SurveyCTO digital platform, which is suitable for designing, monitoring, and archiving surveys (54). Data collection was undertaken using a Samsung Galaxy Tab 2. The survey questionnaire was administered during face-to-face interviews that lasted an average of 30 min. During the fieldwork, the research assistants read out each question word for word and made notes regarding the responses. For open-ended questions, the responses were documented exactly as articulated by the participants. The data archive was encrypted and secured on a server accessible only to the data administrator (first author). To mitigate bias during data collection, the research assistants and first author held regular debriefing sessions and checked protocol compliance every second day of data collection. Daily random checks were also conducted on response patterns, interview, and GIS accuracy.

Community entry was facilitated through the ward executive offices in the sampled areas. We obtained contact information for village/street and sub-village (hamlet) leaders. Prior to the fieldwork, these local leaders who are administratively proximal to households (10-cells and hamlet leaders), in collaboration with community health workers (CHWs), identified the locations of these children and adolescents and connected the research assistants with these households. The identification of households was based on the definition of physical disability provided by the research assistants. The operational definition was broadened to include statements such as “children aged 2 to 18 years with bodily/physical impairment with or without a combination of intellectual or mental impairment” to accurately capture the study population. The research assistants obtained informed consent, screened these children and adolescents for eligibility criteria, and interviewed the carers when the criteria were fulfilled.

### 2.5 Study variables

#### 2.5.1 Dependent variables

In this study there were three dependent variables: psychological wellbeing and symptoms of anxiety and depression. Psychological wellbeing was considered as a continuous outcome, ranging from 0 (the worst possible wellbeing) to 100 (the best possible wellbeing). Symptoms of anxiety and depression were used as binary outcomes: the instrument score ranges from 1 to 4, and an average score for both scales (anxiety and depression separately) of ≥1.75 was considered symptomatic (52, 53). The symptoms of anxiety and depression were labelled 0 (asymptomatic) and 1 (symptomatic).

#### 2.5.2 Independent variables

Sociodemographic factors and caring- and disability-related characteristics were analysed for association with the dependent variables.

### 2.6 Statistical analysis

For the instruments (e.g., the dependent variables), several measures were calculated including Cronbach’s alpha and skewness. For the categorical variables, descriptive statistics were presented with numbers and percentages. Bar graphs were used to show the prevalence of poor psychological wellbeing and symptoms of anxiety and depression. Furthermore, bar graphs together with Pearson’s chi-square tests were used to show the relation between poor psychological wellbeing, anxiety and depression. Multivariable linear and binary logistic regression analyses were used to assess the factors associated with good psychological wellbeing and symptoms of anxiety and depression. First univariable analyses were performed to explore the association between each independent variable and each dependent variable. Variables identified in the univariable analyses with *p*-values of 0.15 or less were included in the multivariable analysis for each outcome. Backwards-elimination procedures were performed until only factors with p-values of less than 0.05 remained in the model for each outcome.

### 2.7 Ethical considerations

The research obtained ethics approval from the institutional review board of the Kilimanjaro Christian Medical University Research Ethics and Review Committee (ID. 2,477). Permission to conduct the study was granted by the executive directors of all involved district councils. The participants were informed about the study, read and signed a consent form to verify their voluntary participation. Confidentiality and anonymity were ensured during data collection, analysis, and result reporting. Research assistants who are physiotherapists were involved to provide a better contextual understanding of the research field and disability-related issues.

## 3 Results

### 3.1 Descriptive characteristics

Table 1 presents descriptive characteristics for the participants. A large majority (91.5%) were female carers, and 41% were aged between 35 and 49. More than half (55%) relied on informal employment, and nearly three quarters (67%) earned approximately $21 USD or less monthly. Most (60%) reported a worse financial state compared to the previous year. The majority reported receiving support from their spouse (59%) and extended family (53%).

**Table 1:** Descriptive characteristics of the study participants (*n* = 212).

Variables	Subcategories	n (%)
**Carer characteristics**		
Age (years)	≤ 34	80 (37.7)
	35–49	87 (41.1)
	≥ 50	45 (21.2)
Sex	Female	194 (91.5)
Education level	No formal education	13 (6.1)
	Secondary level or above	43 (20.3)
	Primary level	156 (73.6)
Marital status	Never married	49 (23.1)
	Married	110 (51.9)
	Widowed	19 (9.0)
	Separated	34 (16.0)
Area of residence	Rural	126 (59.4)
Occupation	Informal employment	116 (54.7)
	Formal employment	10 (4.7)
	Not employed	86 (40.6)
Relationship to child	Mother	158 (74.5)
	Father	14 (6.6)
	Other†	40 (18.9)
Estimated monthly income (USD)	≤ 21	142 (67.0)
	22–64	47 (22.2)
	65 ≥	23 (10.8)
Self-reported financial status compared to previous year	Better	20 (9.4)
	Same	64 (30.2)
	Worse	128 (60.4)
Disruption to social life	All the time	90 (42.5)
	Sometimes	85 (40.1)
	No effect	37 (17.5)
Support from spouse	Absent	88 (41.5)
	Present	124 (58.5)
Support from extended family	Absent	112 (52.8)
	Present	100 (47.2)
Support from NGOs	Absent	91 (42.9)
	Present	121 (57.1)
**Child and adolescent characteristics**		
Age (years)	Children (2–9)	132 (62.3)
	Adolescents (10–18)	80 (37.7)
Sex	Male	102 (48.1)
	Female	110 (51.9)
Diagnosis	Cerebral palsy	142 (67.0)
	Cerebral palsy & other (mixed)	27 (12.7)
	Spina bifida	10 (4.7)
	Muscular dystrophy	12 (5.7)
	Skeletal malformations	9 (4.2)
	Other††	12 (5.7)
Access to rehab services in past month	Yes	77 (36.6)
	Loss of continuous care	84 (39.6)
	Never had	51 (24.1)
Walking limitations	Severe difficulty	124 (58.5)
	Moderate difficulty	64 (30.2)
	No difficulty	24 (11.3)
Participation in schooling	Not possible	27 (12.7)
	Severe difficulty	122 (57.5)
	Moderate difficulty	42 (19.8)
	No difficulty	21 (9.9)

$1 USD ≈ 2,587 Tanzanian Shillings; NGO, non-governmental organisation. ^†^Other: sibling, grandparent, uncle, or aunt. ^††^Other: Down syndrome, amputation, head trauma and spinal cord injury.

Regarding the children’s characteristics, 40% had lost continuity of rehabilitation care, and nearly a quarter (24%) had never received any rehabilitation. More than half of the children had severe walking limitations (59%) and severe difficulties in school participation (58%).

Table 2 presents descriptive measures for the instruments (dependent variables). All three instruments had very good values for Cronbach’s alpha (≥ 0.88) indicating good reliability.

**Table 2:** Descriptive characteristics of the outcome measures (*n* = 212).

Variables	Possible values	Min-max	Mean (Sd)	Md	Skewness (SE)	Cronbach’s alpha	Subcategories	n (%)
Psychological wellbeing; WHO-5 Wellbeing Index	0–100	0–100	51.2 (26.1)	48	0.11 (0.17)	0.94	≤ 50 Poor Wellbeing^1^	107 (50.5)
							> 50	105 (49.5)
Anxiety; HSCL-25	1–4	1–3.8	1.76 (0.68)	1.6	0.92 (0.17)	0.88	< 1.75	123 (58.0)
							≥ 1.75 Symptoms of anxiety^2^	89 (42.0)
Depression; HSCL-25	1–4	1–3.8	1.68 (0.63)	1.5	1.13 (0.17)	0.91	< 1,75	131 (61.8)
							≥ 1.75 Symptoms of depression^2^	81 (38.2)

1 Reference (50).

2 Reference (51).

SD, standard deviation; Md, median; SE, standard error.

For the dependent variable ‘psychological wellbeing’, the test for the normality assumption using skewness found that the data was normally distributed, and then linear regression is an appropriate analysis method.

### 3.2 Prevalence of poor psychological wellbeing and symptoms of anxiety and depression

**Figure 3:**
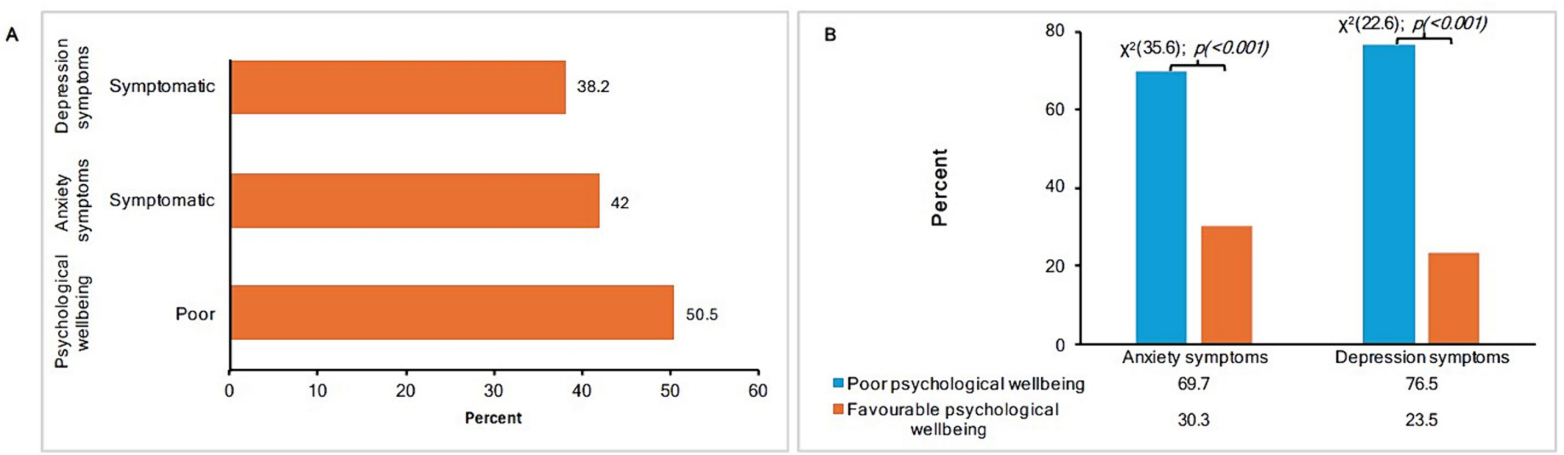
**(A)** The prevalence of poor psychological wellbeing and symptoms of anxiety and depression. **(B)** The association between poor psychological wellbeing and symptoms of anxiety and depression.

Figure 3A shows the prevalence of poor psychological wellbeing and symptoms of anxiety and depression. Of the 212 interviewed carers, more than half (51%) exhibited poor psychological wellbeing, while the prevalence of symptoms of anxiety and depression were 42 and 38%, respectively. Poor psychological wellbeing was significantly associated with symptoms of anxiety and depression, as shown in Figure 3B.

### 3.3 Factors associated with psychological wellbeing and symptoms of anxiety and depression

Tables 3, 4 present the univariable and multivariable regression analyses. A significance threshold of 0.15 was used to determine the inclusion of independent variables for further analysis in the multivariable regression. Thirteen of eighteen explanatory variables for psychological wellbeing met this criterion and were included in the multivariable linear regression. Similarly, 10 independent variables for symptoms of anxiety and nine for symptoms of depression met the predetermined criteria for multivariable logistic regression. Following the backward elimination procedure, the final multivariable models retained six covariates for psychological wellbeing, three for anxiety, and four for depression symptoms.

**Table 3:** The factors associated with psychological wellbeing among carers of children and adolescents with physical disabilities in north-eastern Tanzania (*n* = 212).

Variables	n (%)	Univariable		Multivariable	
		β (95% CI)	*p*-value	aβ (95% CI)	*p*-value
**Age (years)**					
≤ 34	80 (37.7)	8.02 (−1.52, 17.56)	<0.001		
35–49	87 (41.1)	2.05 (−7.52, 11.45)	0.099		
≥ 50	45 (21.2)	Ref			
**Sex**					
Female	194 (91.5)	11.32 (1.28, 23.91)	0.078	11.45 (0.12, 22.85)	0.048
**Education level**					
No formal education	13 (6.1)	12.28 (−2.36, 26.92)	0.100		
Secondary level or above	43 (20.3)	10.64 (1.91, 19.38)	0.017		
Primary level	156 (73.6)	Ref			
**Marital status**					
Married	110 (51.9)	4.06 (−2.99, 11.12)	0.258		
**Area of residence**					
Urban	86 (40.6)	0.88 (−6.32, 8.08)	0.810		
**Occupation**					
Informal employment	116 (54.7)	3.78 (−3.49, 11.04)	0.307		
Formal employment	10 (4.7)	18.07 (1.01, 35.14)	0.038		
Not employed	86 (40.6)	Ref			
**Estimated monthly income (USD)**					
65 ≥	23 (10.8)	22.08 (10.93, 33.23)	<0.001	16.58 (5.95, 27.21)	0.002
22–64	47 (22.2)	9.30 (0.95, 17.64)	0.029	8.50 (0.59, 16.41)	0.035
≤ 21	142 (67.0)	Ref		Ref	
**Self-reported financial status compared to previous year**					
Better	20 (9.4)	13.08 (0.86, 25.30)	0.036		
Same	64 (30.2)	7.60 (−0.19, 15.37)	0.056		
Worse	128 (60.4)	Ref			
**Disruption to social life**					
No	37 (17.5)	24.22 (14.70, 33.75)	<0.001	19.14 (9.27, 29.01)	<0.001
Sometimes	85 (40.1)	6.20 (−1.18, 13.57)	0.099	3.50 (−3.53, 10.53)	0.327
All the time	90 (42.5)	Ref		Ref	
**Support from spouse**					
Present	124 (58.5)	6.95 (−0.17, 14.06)	0.056		
**Support from extended family**					
Present	100 (47.2)	10.06 (3.11, 17.01)	0.005	8.30 (1.94, 14.66)	0.011
**Support from NGOs**					
Present	121 (57.1)	5.76 (−1.34, 12.87)	0.111		
**Child’s age (years)**					
Children (2–9)	132 (62.3)	0.56 (−6.74, 7.85)	0.881		
Adolescents (10–18)	80 (37.7)	Ref			
**Child’s sex**					
Female	109 (51.4)	1.70 (−5.37, 8.78)	0.635		
**Child’s diagnosis**					
Cerebral palsy	142 (67.0)	2.03 (−5.49, 9.55)	0.595		
Other physical disability	70 (33.0)	Ref			
**Access to child rehab services in past month**					
Yes	77 (36.3)	12.48 (3.34, 21.62)	0.008	11.71 (3.15, 20.26)	0.008
Loss of continuous care	84 (39.6)	11.20 (2.21, 20.19)	0.015	9.30 (1.17, 17.43)	0.025
Never had any	51 (24.1)	Ref		Ref	
**Child’s walking limitations**					
No difficulty	24 (11.3)	7.54 (−3.78, 18.86)	0.190		
Moderate difficulty	64 (30.2)	10.72 (2.46, 18.08)	0.010		
Severe difficulty	12 4(58.5)	Ref			
**Child’s participation in schooling**					
No difficulty	21 (9.9)	17.65 (5.76, 29.54)	0.004	13.06 (1.47, 24.66)	0.027
Moderate difficulty	42 (19.8)	10.60 (1.60, 19.60)	0.021	5.35 (−3.25, 13.94)	0.221
Not possible	27 (12.7)	2.22 (−8.49, 12.92)	0.683	−1.27 (−11.18, 8.64)	0.801
Severe difficulty	122 (57.5)	Ref		Ref	

**Table 4:** The factors associated with symptoms of anxiety and depression among carers of children and adolescents with physical disabilities in north-eastern Tanzania (*n* = 212).

Variables	n (%)	Anxiety symptoms (*n* = 89)			Depression symptoms (*n* = 81)		
		cOR (95% CI)	aOR (95% CI)	*p*-value	cOR (95% CI)	aOR (95% CI)	*p*-value
**Age (years)**							
≤ 34	80 (37.7)	0.57 (0.27, 1.21)			0.95 (0.43, 2.10)	1.36 (0.58, 3.20)	0.482
35–49	87 (41.1)	1.28 (0.62, 2.64)			2.06 (1.01, 4.62)**	2.89 (1.26, 6.65)	0.013
≥ 50	45 (21.2)	Ref			Ref	Ref	
**Sex**							
Female	18 (8.5)	1.12 (0.42, 2.95)			1.03 (0.38, 2.78)		
**Education level**							
No formal education	13 (6.1)	0.77 (0.24, 2.45)			0.67 (0.20, 2.28)		
Secondary level or above	43 (20.3)	0.59 (0.29, 1.21)*			o.81 (0.40, 1.64)		
Primary level	156 (73.6)	Ref			Ref		
**Marital status**							
Married	110 (51.9)	0.67 (0.39, 1.16)*			0.67 (0.38, 1.17)		
**Area of residence**							
Urban	86 (40.6)	1.37 (0.79, 2.38)			1.19 (0.68, 2.10)		
**Occupation**							
Informal employment	116 (54.7)	1.42 (0.80, 2.51)			0.82 (0.46, 1,45)		
Formal employment	10 (4.7)	1.13 (0.30, 4.29)			0.35 (0.07, 1.73)		
Not employed	86 (40.6)	Ref			Ref		
**Estimated monthly income (USD)**							
65 ≥	23 (10.8)	0.31 (0.11, 0.88)**			0.18 (0.05, 0.63)**	0.19 (0.05, 0.70)	0.013
22–64	47 (22.2)	0.63 (0.32, 1.25)			0.45 (0.22, 0.93)**	0.41 (0.19, 0.88)	0.022
≤ 21	142 (67.0)	Ref			Ref	Ref	
**Self-reported financial status compared to previous year**							
Better	20 (9.4)	0.32 (0.11, 0.94)**	0.39(0.13, 1.16)	0.090	0.65 (0.24, 1.74)		
Same	64 (30.2)	0.41 (0.22, 0.78)**	0.35(0.18, 0.70)	0.003	0.40 (0.21, 0.78)**		
Worse	128 (60.4)	Ref	Ref		Ref		
**Disruption to social life**							
No	37 (17.5)	0.82 (0.38, 1.77)			0.57 (0.26, 1.28)		
Sometimes	85 (40.1)	0.76 (0.42, 1.39)			0.59 (0.32, 1.09)*		
All the time	90 (42.5)	Ref			Ref		
**Support from spouse**							
Present	124 (58.5)	0.45 (0.26, 0.78)**			0.43 (0.24, 0.75)**	0.44 (0.24, 0.83)	0.010
**Support from extended family**							
Present	100 (47.2)	0.42 (0.24, 0.73)**	0.44(0.25, 0.80)	0.007	0.43 (0.24, 0.77)**	0.51 (0.28, 0.95)	0.035
**Support from NGOs**							
Present	121 (57.1)	0.50 (0.29, 0.87)**			0.56 (0.32, 0.97)**		
**Child’s age (years)**							
Children (2–9)	132 (62.3)	0.50 (0.28, 0.88)**			0.75 (0.42, 1.32)		
Adolescents (10–18)	80 (37.7)	Ref			Ref		
**Child’s sex**							
Female	109 (51.4)	1.24 (0.72, 2.14)			0.83 (0.48, 1.44)		
**Child’s diagnosis**							
Cerebral palsy	142 (67.0)	0.67 (0.38, 1.19)			0.57 (0.32, 1.03)*		
Other physical disability	70 (33.0)	Ref			Ref		
**Access to child rehab services in past month**							
Yes	77 (36.3)	0.37 (0.18, 0.76)**	0.30(0.14, 0.67)	0.003	0.44 (0.21, 0.90)**		
Loss of continuous care	84 (39.6)	0.54 (0.27, 1.10)*	0.54(0.25, 1.13)	0.102	0.56 (0.28, 1.14)*		
Never had any	51 (24.1)	Ref	Ref		Ref		
**Child’s walking limitations**							
No difficulty	24 (11.3)	1.34 (0.56, 3.22)			0.59 (0.23, 1.52)		
Moderate difficulty	64 (30.2)	0.80 (0.43, 1.49)			0.80 (0.43, 1.50)		
Severe difficulty	124 (58.5)	Ref					
**Child’s participation in schooling**							
No difficulty	21 (9.9)	1.64 (0.65, 4.15)			1.28 (0.50, 3.28)		
Moderate difficulty	42 (19.8)	1.01 (0.50, 2.07)			0.86 (0.41, 1.79)		
Not possible	27 (12.7)	1.19 (0.51, 2.76)			1.59 (0.69, 3.68)		
Severe difficulty	122 (57.5)	Ref			Ref		

*p < 0.15, **p < 0.05, ***p < 0.001. cOR, Crude Odds Ratio; NGO, non-governmental organisation; aOR, Adjusted Odds Ratio.

The findings of the multivariable linear regression analysis, presented in Table 3, showed that psychological wellbeing was positively associated with extended-family support (aβ: 8.30, *p* = 0.011), a monthly income of ≥ $65 USD (aβ: 16.58, *p* = 0.002), access to rehabilitation for the child (aβ: 11.71, *p* = 0.008), being a female carer (aβ: 11.48, *p* = 0.008), a lack of disruption to social life (aβ: 19.14, *p* < 0.001), and the child’s participation in school (aβ: 13.06, *p* = 0.027). A monthly income of $22 to $64 USD (aβ: 8.50, *p* = 0.035) was found to be more beneficial than one of ≤ $21 USD.

Table 4 shows that carers who receive support from their spouse (aOR: 0.44, *p* = 0.01) or extended family (aOR: 0.51, *p* = 0.035) were less likely to have symptoms of depression. The same is true for those with a monthly income of ≥ $65 USD (aOR: 0.19, *p* = 0.013), and an income of $22 to $64 USD (aOR: 0.41, *p* = 0.022) as compared to an income of $21 USD or less. Carers aged 35 to 49 are more likely to have symptoms of depression (aOR: 2.89, *p* = 0.013) than those aged 50 and above.

Table 4 also shows that carers with extended-family support (aOR: 0.44, *p* = 0.007), whose child had access to rehabilitation services (aOR: 0.35, *p* = 0.002), and who rated their financial status as the same as the previous year (aOR: 0.35, *p* = 0.003) rather than worse than the previous year were less likely to have symptoms of anxiety.

## 4 Discussion

The rates of symptoms of anxiety and depression in this study are higher than those documented among young men in Dar es Salaam, Tanzania, of 21% for depression and 19% for anxiety (53). In another study, a lower prevalence of 15.5% for both anxiety, and depression was reported in a rural Tanzanian context (55). Despite variability across these populations, the findings of this study are concerning and highlight the urgent need to address the mental health issues faced by carers of children and adolescents with disabilities. Nevertheless, research on the psychological wellbeing and symptoms of anxiety, and depression among carers in Tanzania remains limited. Documenting the prevalence and identifying factors that influence the mental health of carers of children, and adolescents with physical disabilities is essential to fully understand the scope of the issue and to inform public health interventions.

Findings from a systematic review on caregiving in LMICs revealed that psychological, and emotional issues are common among carers, with anxiety and depression being the most prevalent (23). That review reported alarmingly high rates of anxiety and/or depression symptoms, ranging from 52 to 94% (56–58). In Ghana, a higher rate of anxiety (56.2%) and depression (66.2%) has been documented among carers of children with mental health issues, in comparison to the findings of the present study (13). That study also found that female carers are four times more likely to exhibit anxiety symptoms compared to their male counterparts. In the Middle Eastern context, Farajzadeh et al., (14) reported slightly lower rates of anxiety (40.6%) but a slightly higher rate of depression (45%) among carers of children with cerebral palsy during the COVID-19 pandemic. Caregiving responsibilities varies across contexts depending on several factors such as culture, availability and access to resources, the specific needs of those being cared for, and carers intrinsic issues. Differences in mental health burden may be attributed to the complexity of caring which predispose carers to stress and poor psychological wellbeing. This burden is likely to be more severe in resource-poor settings. A distinctive aspect of caring for children and adolescents with physical disabilities is the ongoing need to adjust as the child grows, and the way their disability-related needs evolve. A study conducted in the Kilimanjaro context has shown that, the need to be at home to provide assistance, and physical safety for these vulnerable children can lead to additional stress, predisposing the carers to poor psychological wellbeing (59).

In this study, slightly more than half of the participants had poor psychological wellbeing. Poor psychological wellbeing is commonly reported by carers of children with physical disabilities, and is often accompanied by symptoms of anxiety and depression (29, 60). Furthermore, more than two thirds of the carers with poor psychological wellbeing exhibited high rates of symptoms of depression and anxiety. This trend may reflect the general psychological burden that is closely related to, and often correlates with, anxiety and depression (46, 47, 50). Therefore, it is often recommended that individuals presenting with poor psychological wellbeing undergo further screening for anxiety and/or depression.

Our findings underscore the importance of screening for psychological wellbeing to ensure better mental health for carers. Simple and short screening instruments such as the WHO-5 could be integrated into rehabilitative care in Tanzania in order to detect the early signs of psychological distress among carers. In addition, psychological distress can be attributed to the absence or inadequate resources essential to caring for physically disabled children, and a generally poor socioeconomic situation in this context. This link between poor psychological wellbeing and caring for children with physical disabilities has been reported before (60). Psychological distress and anxiety among carers have also been tied to the societal prejudices and stigmas against children with physical disabilities (61–63).

Although the child’s diagnosis was not significantly associated with any of the outcome variables, it is notable that more than two-thirds (67%) of the children had cerebral palsy. Cerebral palsy is a common neurodevelopmental disability categorised under physical disabilities (64). This condition often presents with multiple comorbidities, necessitating multidisciplinary care and unique resources. In resource-limited settings like Tanzania, the difficulties in accessing the necessary resources for care can contribute to the psychological stress of carers and negatively impact their wellbeing. The severity of the child’s impairment is also a frequently reported factor associated with symptoms of anxiety and depression (16).

The study findings show that psychological wellbeing is significantly better for those carers with less disruption in their social lives. Similarly, carers who feel trapped by the demands of caring for their disabled children also report poorer psychological wellbeing (29). Feeling imprisoned by caregiving responsibilities and losing control over one’s social life can be highly stressful experiences (65). These similarities highlight the complex relationship between the challenges of caring for disabled children and the psychological stress this can cause.

The findings of this study suggest that the overall wellbeing of the disabled child significantly influences that of the carer psychologically. Carers who reported frequent access to rehabilitation care for their physically disabled children had good wellbeing scores compared to those who had never accessed such services. Similarly, the participation of children in school was associated with better psychological wellbeing on the part of the carer than lack of participation in school due to disability-related difficulties. Murphy et al. (29) show that the need to continuously advocate for and ensure the wellbeing of the disabled child is a major source of stress for carers, who must constantly prioritise their children’s needs. Difficulties related to accessing essential rehabilitation and educational services for physically disabled children can pose significant challenges to carers, particularly parents. Similarly, carers of physically disabled children who encounter activity limitations have generally reported poorer health outcomes, including mental health, compared to those of children without disabilities (21). This further suggests the interplay between the child’s wellbeing and the carer’s psychological state.

The findings of this study underscore the importance of supporting carers of physically disabled children in Tanzania. Yamaoka et al. (65) reported that psychological distress was associated with poor social support. The results of the present study have shown that a higher monthly income is associated with good psychological wellbeing and lower likelihood of symptoms of depression. Similar findings have been reported in Tanzania, where emotional support and economic stability were found to be protective against anxiety and depression (26). Conversely, a lower income has been shown to be associated with poor mental health among carers (66). These findings stress the complexity of caring for physically disabled children in resource-constrained settings. Furthermore, it is important to note that a formal and sustainable social-security system for carers who are often poor and live in rural settings is generally lacking in Tanzania. In the absence of this, informal sociocultural support networks are crucial to maintaining the wellbeing of such carers.

In this study, carers who received support from their spouses were 56% less likely to experience symptoms of depression. Extended-family support was another determinant of good psychological wellbeing, and a crucial resource for individuals. The results indicate that the presence of extended**-**family support was associated with an eight-fold increase in scores for psychological wellbeing, as well as reductions of 54 and 49% in the likelihood of experiencing symptoms of anxiety and depression, respectively. In Ghana, Oti-Boadi (67) reported similar findings, whereby support from husbands, friends, the church, and healthcare providers was the core resource for sustaining the mental health of carers. The importance of extended family in this study aligns with the theoretical underpinnings of social capital (68). The concept of family in Tanzania commonly extends beyond the nuclear family (69); in this context, the interactions and networks formed by extended family members serve as important, informal sociocultural support systems for carers of physically disabled children. The study findings illustrate the unique traditional practice of mutual responsibility in challenging situations among extended family members, in this case in relation to caring for physically disabled children.

Research that investigates the factors associated with the psychological wellbeing specifically of carers of children and adolescents with physical disabilities is substantially scarce in Tanzania. Promoting the mental health of these carers is an important public-health concern. In Tanzania, measures to safeguard extended family networks are urgently needed due to the threats of urbanisation and globalisation. Additionally, the integration of screening programmes for mental health in routine care involving carers of physically disabled children is crucial. Introducing a mental-health component for carers during the rehabilitative care of disabled children is recommended. Strengthening mental healthcare in primary healthcare in Tanzania is necessary to promote better mental health for all.

## 5 Strengths and limitations of the study

Our study has both strengths and limitations. It provides valuable quantitative insights into the mental health of carers of children and adolescents with physical disabilities in north-eastern Tanzania. Our survey design ensured a balanced representation, capturing carers with and without access to essential services such as rehabilitation. The findings offer crucial insights for stakeholders involved in the disability agenda in Tanzania, providing information regarding the psychological challenges carers face and suggesting the clear need for measures to protect their mental wellbeing.

However, there are limitations to consider. The assessment of psychological wellbeing, anxiety, and depression relied on self-reported measurements, which can be subjective and prone to recall bias. Additionally, since our study primarily involved carers who were generally the parents of disabled children, it does not fully capture the potential influence of parenthood on psychological wellbeing. The data-collection process was impeded in the Same, Siha, and Mwanga districts by the COVID-19 pandemic, impacting the representation of these geographical areas. Causation could not be established for the reported associations due to the cross-sectional study design. As well, the psychological wellbeing and symptoms of anxiety and depression observed in this study are subject to change over time. Our study focused exclusively on carers of children and adolescents with physical disabilities, which limits the generalisability of the findings to carers of children with other types of disabilities, such as mental and cognitive disabilities. Additionally, there is a possibility that the definition of ‘physical disability’ may have been misunderstood by some, which could have affected the identification of eligible households.

## 6 Conclusion

Carers of children and adolescents with physical disabilities in north-eastern Tanzania face significant mental-health challenges, including poor psychological wellbeing and symptoms of anxiety and depression. Our findings highlight a strong link between the poor psychological wellbeing of carers and symptoms of depression. Support from extended family members plays a crucial role in promoting psychological wellbeing and reducing the risk of symptoms of anxiety and depression. Additionally, the incomes of carers and access to rehabilitation services of physically disabled children positively affects the wellbeing of carers and reduces symptoms of depression. To address the challenges faced by carers, it is essential for the Tanzanian government and civil society to promote cultural values that strengthen extended family networks. Strengthening rehabilitation services through increased governmental funding, particularly in rural and socioeconomically disadvantaged areas, is also important. Furthermore, the integration of mental-health screening in rehabilitation programmes and primary-healthcare services across Tanzania would be beneficial. Regarding financial constraints on healthcare, the government should introduce a free or affordable subscription for children and adolescents with disabilities through the National Insurance fund.

## Data Availability

The raw data supporting the conclusions of this article will be made available by the authors, without undue reservation.
