# Supplementary material for: Psychological wellbeing among carers of children and adolescents with physical disabilities in North-Eastern Tanzania—a cross-sectional survey study
Source: Front Public Health. 2024 Oct 9;12:1437716. doi: 10.3389/fpubh.2024.1437716 (PMC11497636; doi:10.3389/fpubh.2024.1437716)
Supplement: Supplementary file 1 [file Table_1.docx]

Supplementary files

Table S1: The English (original) and Swahili WHO-5 Wellbeing Index.

| s/n | For each of the five statements, please choose the answer that is closest to how you have been feeling over the **LAST TWO WEEKS**  *(Kwa maswali yafuatayo, tutazungumza kuhusu hisia zako kwa muda wa wiki mbili zilizopita.*  ***KWA WIKI MBILI ZILIZOPITA****, ni*  *mara ngapi:)* | All of the time (*Kila Wakati)* | Most of the time (*Mara nyingi)* | More than half of the time  *(Zaidi ya nusu ya wakati)* | Less than half of the time *(Chini ya nusu ya wakati)* | Some of the time (*Wakati mwingine)* | At no time (*Hakuna wakati)* |
| --- | --- | --- | --- | --- | --- | --- | --- |
|  |  | (5) | (4) | (3) | (2) | (1) | (0) |
| 1 | I have felt cheerful and in good spirits.  *(Umejihisi kuwa mchangamfu na mwenye furaha?)* |  |  |  |  |  |  |
| 2 | I have felt calm and relaxed.  *(Umejihisi kuwa mtulivu na aliye pumzika)* |  |  |  |  |  |  |
| 3 | I have felt active and vigorous.  *(Umejihisi kuchangamka na mwenye nguvu?)* |  |  |  |  |  |  |
| 4 | I have woken up feeling fresh and rested.  *(Umeamka ukijihisi mwepesi na aliye pumzika?)* |  |  |  |  |  |  |
| 5 | My daily life has been filled with things that interest me. *(Maisha yako ya kila siku yamejaa vitu vinavyokufurahisha?)* |  |  |  |  |  |  |

**Table S2: The English and Swahili versions of the Hopkins Symptoms Checklist – 25 (HSCL-25)**

| **s/n** | **How much have the following symptoms bothered you during the PAST MONTH?** | **Je katika MWEZI ULIOPITA ikiwemo LEO ni kwa kiasi gani dalili zifuatazo zimekusumbua?** | **Not at all (Hakuna kabisa)** | **A little (Kidogo)** | **Quite a bit (Kiasi)** | **Extremely (Sana)** |
| --- | --- | --- | --- | --- | --- | --- |
|  |  |  | **(1)** | **(2)** | **(3)** | **(4)** |
| 1 | Sudden fear for no reason | Wasiwasi wa ghafla bila sababu |  |  |  |  |
| 2 | Feeling fearful | Kuhisi woga |  |  |  |  |
| 3 | Faintness, dizziness, or weakness | Kupoteza fahamu, kizunguzungu au kukosa nguvu mwilini |  |  |  |  |
| 4 | Nervousness or shakiness inside | Hofu au kutetemeka ndani |  |  |  |  |
| 5 | Heart pounding or racing | Mapigo ya moyo kupiga kwa nguvu au kwenda mbio |  |  |  |  |
| 6 | Trembling | Kutetemeka |  |  |  |  |
| 7 | Feeling tense or keyed up | Kujisikia umekakamaa au una wasiwasi mwingi |  |  |  |  |
| 8 | Headaches | Kichwa kuuma |  |  |  |  |
| 9 | Spells of terror or panic | Vipindi vya hofukuu au kiwewe/ wasiwasi mkubwa |  |  |  |  |
| 10 | Feeling restless, unable to sit still | Hisia za kutokutulia, kukaa sehemu moja |  |  |  |  |
| 11 | Feeling low in energy or slowed down | Kujisikia kukosa nguvu au uvivu |  |  |  |  |
| 12 | Blaming yourself for things | Kujilaumu mwenyewe kuhusiana na mambo Fulani |  |  |  |  |
| 13 | Crying easily | Mwepesi kulia |  |  |  |  |
| 14 | Loss of sexual interest or pleasure | Kukosa hamu ya mapenzi au starehe |  |  |  |  |
| 15 | Poor appetite | Kukosa hamu ya kula |  |  |  |  |
| 16 | Difficulty falling asleep, staying asleep | Kushindwa kupata usingizi, au kuendelea kuwa usingizini |  |  |  |  |
| 17 | Feeling hopeless about the future | Kukosa matumaini kuhusian ana siku zijazo |  |  |  |  |
| 18 | Feeling blue | Kuhisi huzuni |  |  |  |  |
| 19 | Feeling lonely | Kujihisi mpweke |  |  |  |  |
| 20 | Feeling trapped or caught | Mawazo ya kuwa umefungiwa au umekamatwa |  |  |  |  |
| 21 | Worrying too much about things | Kuwa na wasiwasi sana kuhusiana na mambo |  |  |  |  |
| 22 | Feeling no interest in things | Kujisikia kutokuwa na hamu na kitu chochote (vitu ambavyo ni muhimu kwako). |  |  |  |  |
| 23 | Thoughts of ending your life | Mawazo ya kukatisha uhai wako |  |  |  |  |
| 24 | Feeling everything is an effort | Kujisikia hali ya kujilazimisha ili kuweza kufanya kitu chochote. |  |  |  |  |
| 25 | Feelings of worthlessness | Hisia za kutokuwa na thamani |  |  |  |  |
